# 3-Fluoro­anilinium 4-methyl­benzene­sulfonate

**DOI:** 10.1107/S1600536811041055

**Published:** 2011-10-12

**Authors:** Jerry P. Jasinski, James A. Golen, A. S. Praveen, H. S. Yathirajan, B. Narayana

**Affiliations:** a Department of Chemistry, Keene State College, 229 Main Street, Keene, NH 03435-2001, USA; b Department of Studies in Chemistry, University of Mysore, Manasagangotri, Mysore 570 006, India; c Department of Studies in Chemistry, Mangalore University, Mangalagangotri, 574 199, India

## Abstract

In the crystal structure of the title salt, C_6_H_7_FN^+^·C_7_H_7_O_3_S^−^, the components are linked into chains along [010] *via* N—H⋯O hydrogen bonds. Further stabilization is is provided by weak π–π stacking inter­actions, with a centroid–centroid distance of 3.7156 (12) Å.

## Related literature

For mol­ecular salts as solid forms in pharmaceutical formulations, see: Stahl & Wermuth (2002 ▶). For related structures, see: Chanawanno *et al.* (2009 ▶); Chantrapromma *et al.* (2010 ▶); Collier *et al.* (2006 ▶); Fun *et al.* (2010 ▶); Li *et al.* (2005 ▶); Lin (2010 ▶); Tabatabaee & Noozari (2011 ▶); Wu *et al.* (2009 ▶). For standard bond lengths, see: Allen *et al.* (1987 ▶).
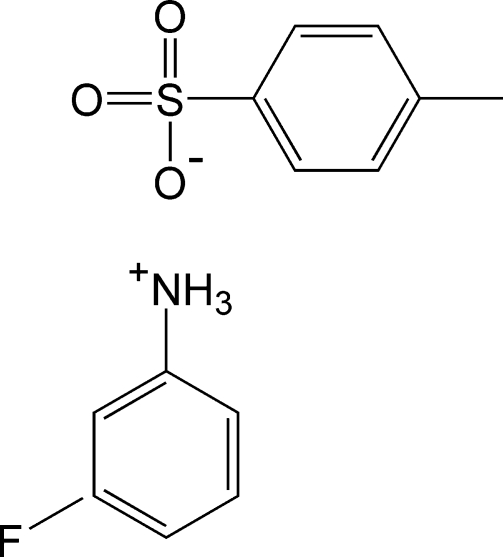


## Experimental

### 

#### Crystal data

C_6_H_7_FN^+^·C_7_H_7_O_3_S^−^
*M* *_r_* = 283.31
Monoclinic, 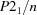

*a* = 14.5385 (7) Å
*b* = 6.4939 (3) Å
*c* = 14.5522 (7) Å
β = 91.429 (4)°
*V* = 1373.47 (11) Å^3^
*Z* = 4
Cu *K*α radiation
μ = 2.25 mm^−1^
*T* = 173 K
0.40 × 0.10 × 0.07 mm

#### Data collection

Oxford Diffraction Xcalibur Eos Gemini diffractometer
Absorption correction: multi-scan (*CrysAlis RED*; Oxford Diffraction, 2010 ▶) *T*_min_ = 0.466, *T*_max_ = 0.858
8663 measured reflections
2642 independent reflections
2076 reflections with *I* > 2σ(*I*)
*R*_int_ = 0.030

#### Refinement

*R*[*F* ^2^ > 2σ(*F* ^2^)] = 0.040
*wR*(*F* ^2^) = 0.121
*S* = 1.05
2642 reflections
174 parameters
H-atom parameters constrained
Δρ_max_ = 0.34 e Å^−3^
Δρ_min_ = −0.34 e Å^−3^

### 

Data collection: *CrysAlis PRO* (Oxford Diffraction, 2010 ▶); cell refinement: *CrysAlis PRO*; data reduction: *CrysAlis RED* (Oxford Diffraction, 2010 ▶); program(s) used to solve structure: *SHELXS97* (Sheldrick, 2008 ▶); program(s) used to refine structure: *SHELXL97* (Sheldrick, 2008 ▶); molecular graphics: *SHELXTL* (Sheldrick, 2008 ▶); software used to prepare material for publication: *SHELXTL*.

## Supplementary Material

Crystal structure: contains datablock(s) global, I. DOI: 10.1107/S1600536811041055/lh5330sup1.cif

Structure factors: contains datablock(s) I. DOI: 10.1107/S1600536811041055/lh5330Isup2.hkl

Supplementary material file. DOI: 10.1107/S1600536811041055/lh5330Isup3.cml

Additional supplementary materials:  crystallographic information; 3D view; checkCIF report

## Figures and Tables

**Table 1:** Hydrogen-bond geometry (Å, °)

*D*—H⋯*A*	*D*—H	H⋯*A*	*D*⋯*A*	*D*—H⋯*A*
N1—H1*NB*⋯O3^i^	0.91	1.89	2.784 (2)	166
N1—H1*NA*⋯O1^ii^	0.91	1.82	2.725 (2)	171
N1—H1*NC*⋯O2	0.91	1.85	2.745 (2)	167

Symmetry codes: (i) 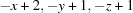; (ii) 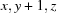.

**Fig. 1.**
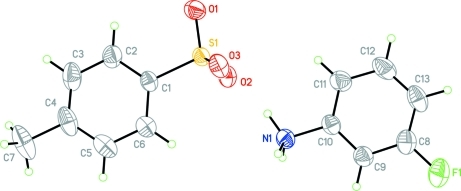
The molecular structure of the title compound showing 50% probability displacement ellipsoids.

**Fig. 2.**
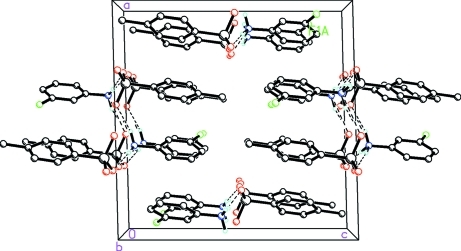
Packing diagram of the title compound viewed along the b axis. Dashed lines indicate N—H···O hydrogen bonds. Only H atoms involved in hydrogen bonds are shown.

## supplementary crystallographic information

### Comment

The importance of molecular salts as solid forms in pharmaceutical formulations is well known (Stahl & Wermuth, 2002). A variety of pharmaceutical drugs are prepared as salts of benzenesulfonic acid and are known as besylates. Benzenesulfonic acid is also used as an acidic catalyst in esterification and dehydration reactions. In the title compound, the proton of the sulfonic group of sulfonic acid has been transferred to the N atom of the 3-fluoroaniline molecule, leading to the formation of the molecular complex, (I). Crystal structures of some benzenesulfonate derivatives, viz., 2,4,6-triamino-1,3,5-triazin-1-ium 4-methylbenzenesulfonate monohydrate (Li *et al.*, 2005), ephedrine besylate (Collier *et al.*, 2006), 2-ethyl-6-methylanilinium 4-methylbenzenesulfonate (Wu *et al.*, 2009), 2-[(E)-2-(4-ethoxyphenyl)ethenyl]-1-methylpyridinium 4-methylbenzenesulfonate monohydrate (Chanawanno *et al.*, 2009), 2-aminopyrimidin-1-ium 4-methylbenzenesulfonate (Tabatabaee & Noozari, 2011), 4-(cyanomethyl)anilinium 4-methylbenzenesulfonate monohydrate (Lin, 2010), 1-methyl-2-[(E)-2-(2-thienyl)etheny] quinolinium 4-bromobenzenesulfonate (Fun *et al.*, 2010) and (E)-2-[4-(dimethylamino)styryl]-1-methylpyridinium 4-methylbenzenesulfonate monohydrate (Chantrapromma *et al.*, 2010) have been reported. In view of the importance of benzenesulphonic acid, we report herein the crystal structure of the title compound (I).

In the crystal structure of the title salt, C_6_H_7_FN^+^, C_7_H_7_O_3_S^-^, (Fig. 1) N—H···O hydrogen bonds link the components into one-dimensional chains along [010] (Fig. 2).  Further stabilization is is provided by weak π–π stacking interactions with a centroid to centroid distance of 3.7156 (12)Å.

### Experimental

4-methylbenzenesulfonic acid monohydrate (1 g, 5.25 mmol) was added to a stirred solution of 3-fluoroaniline (0.58 g, 5.25 mmol ) in methanol (10 mL). Resulting mixture was stirred at 323 K for 10 minutes and cooled to room temperature to obtain the title compound (I), Fig. 1. The single crystal was grown from methanol by slow evaporation method (m.p.: 533 K).

### Refinement

H1NA, H1NB and H1NC were intially located in a difference Fourier map. These and all of the remaining H atoms were placed in their calculated positions and then refined using the riding model with Atom—H lengths of 0.91Å (NH), 0.95Å (CH) or 0.98Å (CH_3_). Isotropic displacement parameters for these atoms were set to 1.20 (CH, NH) or 1.50 (CH_3_) times *U*_eq_ of the parent atom.

### Crystal data

**Table:**

C_6_H_7_FN^+^·C_7_H_7_O_3_S^−^	*F*(000) = 592
*M**_r_* = 283.31	*D*_x_ = 1.370 Mg m^−^^3^
Monoclinic, *P*2_1_/*n*	Cu *K*α radiation, λ = 1.54178 Å
Hall symbol: -P 2yn	Cell parameters from 2843 reflections
*a* = 14.5385 (7) Å	θ = 4.2–71.3°
*b* = 6.4939 (3) Å	µ = 2.25 mm^−^^1^
*c* = 14.5522 (7) Å	*T* = 173 K
β = 91.429 (4)°	Rod, colorless
*V* = 1373.47 (11) Å^3^	0.40 × 0.10 × 0.07 mm
*Z* = 4	

### Data collection

**Table:**

Oxford Diffraction Xcalibur Eos Gemini diffractometer	2642 independent reflections
Radiation source: Enhance (Cu) X-ray Source	2076 reflections with *I* > 2σ(*I*)
graphite	*R*_int_ = 0.030
Detector resolution: 16.1500 pixels mm^-1^	θ_max_ = 71.5°, θ_min_ = 4.3°
ω scans	*h* = −17→17
Absorption correction: multi-scan (*CrysAlis RED*; Oxford Diffraction, 2010)	*k* = −7→7
*T*_min_ = 0.466, *T*_max_ = 0.858	*l* = −13→17
8663 measured reflections	

### Refinement

**Table:**

Refinement on *F*^2^	Primary atom site location: structure-invariant direct methods
Least-squares matrix: full	Secondary atom site location: difference Fourier map
*R*[*F*^2^ > 2σ(*F*^2^)] = 0.040	Hydrogen site location: inferred from neighbouring sites
*wR*(*F*^2^) = 0.121	H-atom parameters constrained
*S* = 1.05	*w* = 1/[σ^2^(*F*_o_^2^) + (0.0665*P*)^2^ + 0.2763*P*] where *P* = (*F*_o_^2^ + 2*F*_c_^2^)/3
2642 reflections	(Δ/σ)_max_ < 0.001
174 parameters	Δρ_max_ = 0.34 e Å^−^^3^
0 restraints	Δρ_min_ = −0.34 e Å^−^^3^

### Special details

**Table:**

Geometry. All esds (except the esd in the dihedral angle between two l.s. planes) are estimated using the full covariance matrix. The cell esds are taken into account individually in the estimation of esds in distances, angles and torsion angles; correlations between esds in cell parameters are only used when they are defined by crystal symmetry. An approximate (isotropic) treatment of cell esds is used for estimating esds involving l.s. planes.
Refinement. Refinement of *F*^2^ against ALL reflections. The weighted *R*-factor *wR* and goodness of fit *S* are based on *F*^2^, conventional *R*-factors *R* are based on *F*, with *F* set to zero for negative *F*^2^. The threshold expression of *F*^2^ > σ(*F*^2^) is used only for calculating *R*-factors(gt) *etc*. and is not relevant to the choice of reflections for refinement. *R*-factors based on *F*^2^ are statistically about twice as large as those based on *F*, and *R*- factors based on ALL data will be even larger.

### Fractional atomic coordinates and isotropic or equivalent isotropic displacement parameters (Å^2^)

**Table:**

	*x*	*y*	*z*	*U*_iso_*/*U*_eq_	
S1	0.84338 (3)	0.25122 (6)	0.46398 (3)	0.03256 (17)	
F1	0.93374 (13)	1.1392 (2)	0.84280 (10)	0.0794 (5)	
O1	0.79440 (10)	0.0607 (2)	0.48237 (9)	0.0439 (4)	
O2	0.79079 (9)	0.4330 (2)	0.48854 (9)	0.0412 (3)	
O3	0.93526 (10)	0.2539 (2)	0.50709 (9)	0.0416 (3)	
N1	0.88997 (11)	0.7501 (2)	0.56685 (11)	0.0366 (4)	
H1NC	0.8610	0.6332	0.5475	0.044*	
H1NB	0.9504	0.7439	0.5524	0.044*	
H1NA	0.8634	0.8611	0.5387	0.044*	
C1	0.85900 (13)	0.2623 (3)	0.34404 (12)	0.0338 (4)	
C2	0.83246 (14)	0.1018 (4)	0.28787 (14)	0.0462 (5)	
H2A	0.8042	−0.0165	0.3132	0.055*	
C3	0.84700 (15)	0.1129 (4)	0.19455 (15)	0.0559 (6)	
H3A	0.8291	0.0007	0.1562	0.067*	
C4	0.88712 (14)	0.2840 (4)	0.15601 (14)	0.0527 (6)	
C5	0.91260 (15)	0.4458 (4)	0.21321 (15)	0.0510 (6)	
H5A	0.9398	0.5652	0.1876	0.061*	
C6	0.89915 (14)	0.4368 (3)	0.30711 (14)	0.0439 (5)	
H6A	0.9172	0.5485	0.3457	0.053*	
C7	0.90233 (19)	0.2933 (5)	0.05369 (16)	0.0751 (9)	
H7A	0.8498	0.2305	0.0208	0.113*	
H7B	0.9586	0.2180	0.0393	0.113*	
H7C	0.9084	0.4372	0.0346	0.113*	
C8	0.90357 (16)	0.9645 (3)	0.80119 (15)	0.0486 (5)	
C9	0.91293 (14)	0.9489 (3)	0.70742 (13)	0.0410 (5)	
H9A	0.9395	1.0566	0.6727	0.049*	
C10	0.88200 (13)	0.7699 (3)	0.66649 (13)	0.0349 (4)	
C11	0.84357 (14)	0.6132 (3)	0.71594 (14)	0.0461 (5)	
H11A	0.8232	0.4906	0.6862	0.055*	
C12	0.83496 (15)	0.6368 (4)	0.81015 (15)	0.0529 (6)	
H12A	0.8081	0.5297	0.8450	0.064*	
C13	0.86476 (15)	0.8130 (4)	0.85343 (14)	0.0506 (5)	
H13A	0.8587	0.8299	0.9178	0.061*	

### Atomic displacement parameters (Å^2^)

**Table:**

	*U*^11^	*U*^22^	*U*^33^	*U*^12^	*U*^13^	*U*^23^
S1	0.0320 (3)	0.0375 (3)	0.0283 (3)	−0.00209 (16)	0.00377 (18)	−0.00070 (17)
F1	0.1225 (14)	0.0660 (10)	0.0500 (8)	−0.0095 (9)	0.0073 (8)	−0.0225 (7)
O1	0.0454 (8)	0.0452 (8)	0.0413 (7)	−0.0080 (6)	0.0032 (6)	0.0051 (6)
O2	0.0413 (8)	0.0467 (8)	0.0359 (7)	0.0036 (6)	0.0064 (6)	−0.0057 (6)
O3	0.0361 (8)	0.0580 (9)	0.0309 (7)	−0.0023 (6)	0.0018 (6)	0.0006 (6)
N1	0.0354 (9)	0.0401 (8)	0.0345 (8)	−0.0007 (6)	0.0065 (7)	−0.0015 (6)
C1	0.0289 (9)	0.0441 (10)	0.0286 (9)	0.0011 (7)	0.0028 (7)	−0.0005 (7)
C2	0.0391 (11)	0.0579 (12)	0.0416 (11)	−0.0123 (9)	0.0038 (9)	−0.0111 (9)
C3	0.0408 (12)	0.0859 (17)	0.0411 (11)	−0.0093 (11)	0.0022 (9)	−0.0214 (11)
C4	0.0326 (11)	0.0932 (18)	0.0324 (11)	0.0096 (11)	0.0018 (9)	−0.0031 (11)
C5	0.0454 (13)	0.0633 (14)	0.0448 (12)	0.0046 (10)	0.0098 (10)	0.0135 (10)
C6	0.0460 (12)	0.0467 (11)	0.0393 (10)	−0.0014 (9)	0.0062 (9)	0.0007 (9)
C7	0.0528 (15)	0.139 (3)	0.0339 (12)	0.0110 (16)	0.0056 (11)	0.0037 (14)
C8	0.0513 (13)	0.0533 (12)	0.0415 (11)	0.0046 (10)	0.0039 (9)	−0.0089 (9)
C9	0.0455 (12)	0.0405 (10)	0.0375 (10)	0.0011 (8)	0.0073 (8)	−0.0006 (8)
C10	0.0283 (9)	0.0433 (10)	0.0334 (9)	0.0024 (7)	0.0067 (7)	0.0001 (7)
C11	0.0401 (11)	0.0542 (12)	0.0440 (11)	−0.0093 (9)	0.0037 (9)	0.0036 (9)
C12	0.0404 (12)	0.0742 (16)	0.0447 (12)	−0.0089 (11)	0.0100 (9)	0.0139 (11)
C13	0.0400 (11)	0.0786 (15)	0.0338 (10)	0.0085 (11)	0.0091 (9)	0.0028 (10)

### Geometric parameters (Å, °)

**Table:**

S1—O1	1.4555 (14)	C5—C6	1.386 (3)
S1—O2	1.4561 (14)	C5—H5A	0.9500
S1—O3	1.4615 (15)	C6—H6A	0.9500
S1—C1	1.7671 (18)	C7—H7A	0.9800
F1—C8	1.354 (3)	C7—H7B	0.9800
N1—C10	1.463 (2)	C7—H7C	0.9800
N1—H1NC	0.9100	C8—C13	1.373 (3)
N1—H1NB	0.9100	C8—C9	1.378 (3)
N1—H1NA	0.9100	C9—C10	1.376 (3)
C1—C2	1.374 (3)	C9—H9A	0.9500
C1—C6	1.389 (3)	C10—C11	1.373 (3)
C2—C3	1.381 (3)	C11—C12	1.388 (3)
C2—H2A	0.9500	C11—H11A	0.9500
C3—C4	1.381 (3)	C12—C13	1.371 (3)
C3—H3A	0.9500	C12—H12A	0.9500
C4—C5	1.385 (3)	C13—H13A	0.9500
C4—C7	1.512 (3)		
			
O1—S1—O2	112.44 (8)	C5—C6—C1	119.1 (2)
O1—S1—O3	112.18 (8)	C5—C6—H6A	120.4
O2—S1—O3	111.39 (8)	C1—C6—H6A	120.4
O1—S1—C1	106.95 (8)	C4—C7—H7A	109.5
O2—S1—C1	106.89 (8)	C4—C7—H7B	109.5
O3—S1—C1	106.56 (8)	H7A—C7—H7B	109.5
C10—N1—H1NC	109.5	C4—C7—H7C	109.5
C10—N1—H1NB	109.5	H7A—C7—H7C	109.5
H1NC—N1—H1NB	109.5	H7B—C7—H7C	109.5
C10—N1—H1NA	109.5	F1—C8—C13	119.1 (2)
H1NC—N1—H1NA	109.5	F1—C8—C9	117.7 (2)
H1NB—N1—H1NA	109.5	C13—C8—C9	123.2 (2)
C2—C1—C6	120.20 (18)	C10—C9—C8	116.80 (19)
C2—C1—S1	121.05 (15)	C10—C9—H9A	121.6
C6—C1—S1	118.74 (14)	C8—C9—H9A	121.6
C1—C2—C3	119.9 (2)	C11—C10—C9	122.15 (19)
C1—C2—H2A	120.1	C11—C10—N1	119.83 (17)
C3—C2—H2A	120.1	C9—C10—N1	118.02 (16)
C4—C3—C2	121.2 (2)	C10—C11—C12	118.9 (2)
C4—C3—H3A	119.4	C10—C11—H11A	120.5
C2—C3—H3A	119.4	C12—C11—H11A	120.5
C3—C4—C5	118.42 (19)	C13—C12—C11	120.7 (2)
C3—C4—C7	120.4 (2)	C13—C12—H12A	119.7
C5—C4—C7	121.2 (2)	C11—C12—H12A	119.7
C4—C5—C6	121.2 (2)	C12—C13—C8	118.23 (19)
C4—C5—H5A	119.4	C12—C13—H13A	120.9
C6—C5—H5A	119.4	C8—C13—H13A	120.9
			
O1—S1—C1—C2	−4.3 (2)	C4—C5—C6—C1	−0.3 (3)
O2—S1—C1—C2	−124.97 (17)	C2—C1—C6—C5	−0.5 (3)
O3—S1—C1—C2	115.82 (18)	S1—C1—C6—C5	179.29 (16)
O1—S1—C1—C6	175.90 (15)	F1—C8—C9—C10	179.88 (19)
O2—S1—C1—C6	55.27 (17)	C13—C8—C9—C10	−0.7 (3)
O3—S1—C1—C6	−63.94 (17)	C8—C9—C10—C11	0.0 (3)
C6—C1—C2—C3	1.0 (3)	C8—C9—C10—N1	179.24 (17)
S1—C1—C2—C3	−178.81 (17)	C9—C10—C11—C12	0.5 (3)
C1—C2—C3—C4	−0.7 (4)	N1—C10—C11—C12	−178.71 (18)
C2—C3—C4—C5	−0.1 (3)	C10—C11—C12—C13	−0.4 (3)
C2—C3—C4—C7	−179.9 (2)	C11—C12—C13—C8	−0.3 (3)
C3—C4—C5—C6	0.6 (3)	F1—C8—C13—C12	−179.7 (2)
C7—C4—C5—C6	−179.6 (2)	C9—C8—C13—C12	0.8 (3)

### Hydrogen-bond geometry (Å, °)

**Table:**

*D*—H···*A*	*D*—H	H···*A*	*D*···*A*	*D*—H···*A*
N1—H1NB···O3^i^	0.91	1.89	2.784 (2)	166.
N1—H1NA···O1^ii^	0.91	1.82	2.725 (2)	171.
N1—H1NC···O2	0.91	1.85	2.745 (2)	167.

Symmetry codes: (i) −*x*+2, −*y*+1, −*z*+1; (ii) *x*, *y*+1, *z*.
